# Diabetic Macular Edema in Maintenance Intravitreal Scheduling

**DOI:** 10.3390/pharmaceutics17050605

**Published:** 2025-05-02

**Authors:** Enzo Maria Vingolo, Mattia Calabro, Simona Mascolo, Filippo Miccichè, Lorenzo Casillo, Stefano Lupo, Feliciana Menna

**Affiliations:** U.O.C. Ophthalmology, Polo Pontino-Ospedale A. Fiorini, Sense Organs Department, University Sapienza of Rome, 04019 Terracina, Italy; enzomaria.vingolo@uniroma1.it (E.M.V.);

**Keywords:** central retinal thickness (CRT), cystoid center-involving diabetic macular edema (DME), best-corrected visual acuity (BCVA), diabetic retinopathy (DR), vascular endothelial growth factor (VEGF), spectral-domain optical coherence tomography (SD-OCT)

## Abstract

**Objectives:** This study aimed to assess the impact of an oral formulation combining bromelain (250 mg) derived from *Ananas comosus* (L.) Merr. Jambs and diosmin mcgSMIN Plus (250 mg) on visual acuity and central retinal thickness (CRT) in patients undergoing anti-VEGF intravitreal maintenance therapy (IVT) for cystoid, center-involving diabetic macular edema (DME). **Methods:** A total of forty patients with type 2 diabetes and center-involving DME, all receiving anti-VEGF maintenance therapy, were prospectively enrolled in a case-control study. Group A (20 eyes) was administered one tablet containing 250 mg of bromelain and 250 mg of diosmin mcgSMIN Plus twice daily for a period of two months. Group B (20 eyes) was monitored without drug administration until the next IVT. After two months and IVT administration, the groups were switched. At the end of the study, the collected data were divided into two groups. The treatment group included data from patients who received the dietary supplement, and the control group consisted of data from patients who were only observed during anti-VEGF therapy. Best-corrected visual acuity (BCVA) and CRT measurements were assessed at baseline, after two months, and after four months. **Results:** A statistically significant improvement in CRT was observed in the treatment group compared to the control group during follow-up (*p* < 0.05). However, the improvement in BCVA was not statistically significant. **Conclusions:** Orally administered combination of bromelain 250 mg and 250 mg diosmin mcgSMIN Plus has positive impact on central retinal thickness in patients treated for cystoid center-involving diabetic macular edema (DME) with anti-VEGF intravitreal maintenance therapy.

## 1. Introduction

Diabetic retinopathy (DR) represents one of the most frequent microvascular complications associated with diabetes mellitus and remains a primary cause of vision loss and blindness on a global scale. With the growing prevalence of diabetes, the incidence and burden of DR continue to increase, impacting millions worldwide.

Several features make the retina particularly vulnerable in diabetes, including its low-oxygen environment [1], high metabolic requirements [2], and the intricate structure of the blood–retinal barrier [3,4]. Progressive injury to the retinal microvasculature results in capillary closure, ischemia, and increased vascular permeability. These pathological processes give rise to two major vision-threatening outcomes: proliferative diabetic retinopathy (PDR) and diabetic macular edema (DME) [5].

The pathogenesis of DME is complex and involves multiple mechanisms, such as chronic inflammation, oxidative stress, and the upregulation of vascular endothelial growth factor (VEGF). VEGF plays a critical role by promoting vascular permeability, resulting in the accumulation of fluid within the retinal layers and the development of macular edema [6]. Accordingly, therapies that inhibit VEGF have become a cornerstone in the management of DME.

Over the last two decades, significant advancements have been made in the management of DME. Intravitreal pharmacological therapies, particularly anti-VEGF agents such as bevacizumab, ranibizumab, aflibercept, and faricimab, have transformed treatment paradigms. These therapies have demonstrated superior visual outcomes compared to traditional laser therapy [7,8]. Anti-VEGF medications act by inhibiting VEGF-induced vascular permeability, effectively reducing macular edema and improving vision. Nevertheless, frequent intravitreal injections and variability in patient responses present ongoing challenges.

For patients who do not respond adequately to anti-VEGF therapy, corticosteroid implants such as dexamethasone (Ozurdex^®^) and fluocinolone acetonide (Iluvien^®^) provide an alternative approach by modulating inflammatory pathways and stabilizing the blood–retinal barrier [7,9]. However, their use is associated with potential ocular side effects, such as cataract formation and elevated intraocular pressure [10]. Furthermore, the IVT route carries inherent risks for endophthalmitis, a severe and vision-threatening infection [11]. Therefore, strategies that could reduce the number of IVT injections, such as adjunctive oral therapies, are of clinical interest for minimizing procedural risks while maintaining therapeutic efficacy.

In addition to conventional treatments, there is growing interest in the influence of systemic and nutritional factors on DME management. In particular, nutritional supplements—such as antioxidants (vitamins C and E, lutein, and zeaxanthin), omega-3 fatty acids, fenofibric acid, and vitamin D—have been explored for their potential to reduce oxidative stress and inflammation, which play critical roles in DME progression [9,12,13,14]. While some studies suggest that dietary modifications and targeted supplementation may support retinal health, more extensive clinical trials are required to confirm their effectiveness in managing DME [15].

This study investigates the potential role of a combination of bromelain (250 mg) and diosmin mcgSMIN Plus (250 mg) in managing diabetic retinopathy and DME, focusing on their anti-inflammatory and vascular-stabilizing effects.

The subsequent sections present clinical outcomes and retinal morphological changes observed during this study.

## 2. Materials and Methods

Forty eyes of forty patients with type 2 diabetes and cystoid diabetic macular edema, treated with a maintenance protocol based on anti-VEGF, were randomly selected and enrolled in this prospective case-control study. Only eyes that had completed the loading phase and were on a maintenance protocol based on bi-monthly injections were included. All patients underwent a complete ophthalmological examination, including BCVA, anterior segment slit-lamp and fundus examination, measurement of central retinal thickness (CRT), and fundus photography by spectral-domain optical coherence tomography (SD-OCT) at the U.O.C. Ophthalmology, Polo Pontino-Ospedale A. Fiorini, Terracina (LT), Italy.

Informed consent was obtained from all participants, and the study was conducted in compliance with the principles of the Helsinki Declaration of 1964 and its later revisions.

The classification of DR severity was carried out according to the International Clinical Diabetic Retinopathy and Diabetic Macular Edema Severity Scale. Eligibility criteria included (a) male and female patients aged between 60 and 90 years; (b) individuals with type 2 diabetes managed through oral medications or subcutaneous anti-hyperglycemic therapy; (c) a diabetes duration ranging from 5 to 15 years; (d) diagnosis of non-proliferative diabetic retinopathy, evidenced by the presence of microaneurysms; (e) confirmation of diabetes mellitus based on the American Diabetes Association standards; (f) best-corrected visual acuity (BCVA) of at least 20/50, measured using ETDRS charts; (g) absence of systemic complications related to diabetes; (h) confirmed cystoid center-involving DME following completion of the anti-VEGF loading phase; (i) central retinal thickness (CRT) ranging from 200 µm to 600 µm.

Exclusion criteria included (a) proliferative diabetic retinopathy; (b) history of hypersensitivity to study medications; (c) macular pathologies unrelated to diabetes (e.g., foveal scars, exudative macular degeneration, uveitis, or epiretinal membranes); (d) less than 20 weeks of intravitreal treatment; (e) more than 36 weeks of intravitreal treatment; (f) cataract surgery within 12 months; (g) prior ocular surgeries within 12 months. Mild DME was defined as central retinal thickness (CRT) below 400 µm, following the guidelines of the National Institute for Health and Care Excellence (NICE; accessed 3 January 2025).

The sample size was calculated using MedCalc version 10.0 (MedCalc, Ostend, Mariakerke, Belgium). The minimum sample size requirement for a t-test with an alpha level of 0.05, and a power of 0.8, was calculated to be 20 for each group.

The study design chosen was a cross-over model in which all patients received the drug and the washout period. After a two-month washout corresponding to the interval between IVT administrations, Group “A” received one pill twice a day for two months, and after two months, all examinations were repeated. Group “B,” after the washout, was only monitored without drug administration until the next IVT. The washout period corresponded to the interval between scheduled anti-VEGF intravitreal injections (approximately two months). This timing was selected based on clinical practice to minimize potential carryover effects of the oral supplement; however, residual effects cannot be entirely ruled out.

After two months and the subsequent IVT administration, the groups were crossed over following the clinical study design to minimize intraindividual variability. Group “A” patients were thereafter monitored without additional treatment, whereas Group “B” patients began receiving the same oral regimen for two months. Upon study completion, the collected data were reorganized into two distinct groups: a treatment group (20 eyes) and a control group (20 eyes). The treatment group comprised data from periods when patients received the dietary supplement (Redemase 500^®^, Ophthagon Srl, Rome, Italy), consisting of bromelain (250 mg) and diosmin mcgSMIN Plus (250 mg), administered as two tablets daily before meals over a continuous four-month interval. In contrast, the control group data reflected the periods when patients were monitored solely under anti-VEGF maintenance therapy, without any oral supplementation.

Ophthalmic assessments—including BCVA, anterior segment slit-lamp examination, fundus examination, CRT measurement, and fundus photography—were performed at baseline and at two- and four-month intervals using a SD-OCT instrument (Spectralis, Heidelberg Engineering).

A sub-analysis was performed within the treatment group to assess individual responses to therapy. Patients achieving a reduction greater than 30 µm in CRT were classified as good responders, whereas those whose CRT remained stable or increased relative to baseline were considered poor responders.

### 2.1. Scanning Protocol

One eye per patient was included in the analysis, and the highest-quality scans were selected for evaluation. The OCT acquisition protocol consisted of a macular line scan and a 6 × 6 mm macular cube, centered on the fovea. All scans were reviewed by an expert ophthalmologist (F.M.) to ensure proper segmentation accuracy.

### 2.2. Statistical Analysis

Statistical analyses were carried out using Prism software, version 9.5.0 (GraphPad Software Inc., San Diego, CA, USA). Data were expressed as mean ± standard deviation (SD) for variables with normal distribution, and as median with interquartile range (IQR) for those with non-normal distribution. Normality was evaluated using the Anderson–Darling and Kolmogorov–Smirnov tests. Based on the distribution pattern, comparisons between groups were performed using either the Student’s *t*-test or the Mann–Whitney U-test. Changes in CRT and BCVA over time were analyzed through a mixed-design ANOVA. A *p*-value below 0.05 was considered indicative of statistical significance.

## 3. Results

The study included 40 eyes from 40 patients (16 males and 24 females), with a mean age of 70 ± 6.77 years, all diagnosed with mild non-proliferative DR and cystoid center-involving DME. Participants were randomly assigned into two groups: the treatment group (Group A, *n* = 20 eyes) and the control group (Group B, *n* = 20 eyes). Key demographic and clinical characteristics are presented in Table 1.

**Figure 1:**
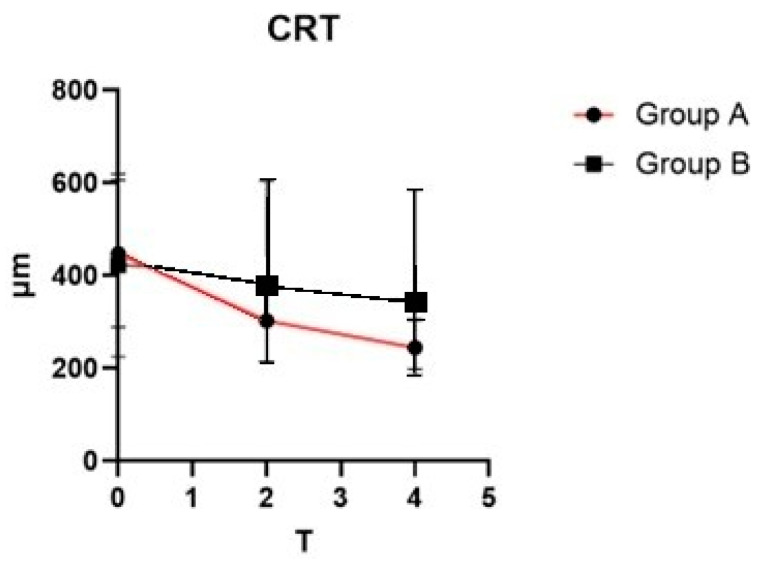
Central retinal thickness (CRT) changes over time in groups A and B.

At baseline, no statistically significant differences were identified between the two groups concerning demographic characteristics and ocular parameters, including BCVA and CRT. Analysis through mixed-model ANOVA revealed a significant interaction between time and treatment effect on CRT, with F (2, 114) = 5.645 (*p* = 0.0046). A noticeable reduction in CRT was observed in Group A over the study period (Figure 1). In contrast, Group B demonstrated only a minimal downward trend in CRT measurements across follow-up evaluations.

Conversely, the interaction between time and treatment was not significant in BCVA with F (2, 114) = 0.929 (*p* = 0.3976), suggesting that structural improvements may not always translate into immediate functional visual gains, possibly due to chronic retinal changes or retinal ischemia.

Among the forty eyes evaluated, twenty-one were pseudophakic (Group A: eleven eyes; Group B: ten eyes), while ten exhibited N2C3 cataracts (Group A: five eyes; Group B: five eyes), five presented with N3C2 cataracts (Group A: three eyes; Group B: two eyes), and four had N4C3 cataracts (Group A: two eyes; Group B: two eyes). No adverse effects were reported in Group A, which received the oral supplementation of the fixed combination of bromelain (250 mg) and diosmin mcgSMIN Plus (250 mg). Regular fundus examinations were performed at each follow-up visit, and no vitreoretinal interface abnormalities were identified. Although no adverse events were reported in the treatment group, systematic safety monitoring included scheduled slit-lamp examination, fundus examination, intraocular pressure measurement, and patient-reported outcome evaluations to capture any potential systemic side effects such as gastrointestinal symptoms or allergic reactions.

## 4. Discussion

Diabetic macular edema (DME) is a major cause of vision loss in patients with diabetes, resulting from the accumulation of fluid in the macula due to breakdown of the blood–retinal barrier. DME often occurs in conjunction with diabetic retinopathy and can lead to significant visual impairment if left untreated [5]. Current treatment strategies primarily involve intravitreal anti-VEGF injections, which aim to reduce retinal edema and improve visual acuity. Additionally, other treatment options, such as intravitreal corticosteroids and laser photocoagulation, are commonly used [8].

OCT, alongside visual acuity assessments, plays a key role in the diagnosis and management of DME [16]. Several OCT-derived biomarkers have shown potential correlations with visual recovery and CRT, including the extent and amount of intraretinal fluid (IRF), subretinal fluid (SRF), preservation of the external limiting membrane (ELM), the number of hyperreflective foci (HRF) [17], and the presence of disorganization of retinal inner layers (DRIL), characterized by the loss of clear boundaries between the ganglion cell layer, inner plexiform layer, inner nuclear layer, and outer plexiform layer [18]. Additionally, the percentage of disruption in the photoreceptor inner segment/outer segment (IS/OS) junction is considered an important marker [19].

The development and advancement of diabetic retinopathy and DME are attributed not only to vascular damage resulting in hematoretinal barrier breakdown but also to dysfunction within the neurovascular unit [20].

Neurovascular units are defined as neurons (ganglion cells, amacrine cells, horizontal cells, and bipolar cells), glial cells (Müller cells, microglia, and astrocytes), and vessels (endothelial cells, pericytes, and basement membrane). Among the microglia, two subtypes are distinguished: a pro-inflammatory one (M1) and an anti-inflammatory one (M2) [21].

Under conditions of hyperglycemic stress, microglia switch to the proinflammatory M1 stage, with production of TNF-α, IL-6, MCP-1, and VEGF via the ERK1/2-NFkb signaling pathway [22,23]. Through paracrine mediation, Müller cells and astrocytes are also activated and amplify inflammatory responses by producing proinflammatory cytokines [24].

This new knowledge of neuroinflammation in diabetic macular edema has brought increasing interest in the use of oral supplements or adjunctive therapies to help manage DME, particularly in reducing inflammation and improving vascular health [9]. These therapies aim to complement primary treatments, potentially improving outcomes between injections or reducing the frequency of intravitreal injections.

Various natural compounds have been studied for their potential benefits in managing DME, including flavonoids, antioxidants, and enzymes such as bromelain, all of which have been shown to possess anti-inflammatory and edematous properties [12,13,14].

Our study focused on the potential role of a fixed combination of bromelain (250 mg) and diosmin mcgSMIN Plus (250 mg) in the management of cystoid center-involving DME.

Bromelain, a proteolytic enzyme extracted from pineapple (*Ananas comosus*), has been shown to exhibit anti-inflammatory, antithrombotic, and fibrinolytic activities. These properties are particularly relevant in DME, where chronic inflammation and microvascular dysfunction contribute to disease progression. Bromelain has demonstrated the ability to inhibit the Raf-1/extracellular-regulated-kinase-(ERK-) 2 pathways of T cells, which are involved in VEGF production [25]. Bromelain has also been reported to inhibit pro-inflammatory cytokines such as TNF-α and interleukins, which are implicated in retinal vascular permeability [26]. Furthermore, bromelain’s ability to reduce blood viscosity and platelet aggregation may help improve retinal microcirculation in neurovascular units, thereby decreasing ischemic damage in diabetic retinopathy [27,28].

Diosmin, a flavonoid predominantly sourced from citrus fruits, is extensively utilized for managing chronic venous insufficiency because of its vasoprotective and anti-edematous properties. It has been shown to reinforce tight junctions, uphold the integrity of the blood–retinal barrier, and decrease retinal vascular permeability during ischemia/reperfusion events in the retina [29]. Preclinical research has indicated that diosmin provides antioxidant protection, safeguarding retinal endothelial cells from oxidative stress-related injury [30]. Moreover, its capacity to regulate VEGF expression and inflammatory cytokine activity points toward a potential synergistic effect when combined with anti-VEGF therapies in the management of DME [31,32].

In our study, we found that patients who received a fixed combination of bromelain (250 mg) and diosmin mcgSMIN Plus (250 mg) demonstrated an improvement in retinal edema and a reduction in CRT. The study population was restricted to elderly patients (60–90 years) with mild non-proliferative diabetic retinopathy and cystoid center-involving DME, excluding individuals with proliferative diabetic retinopathy or other ocular comorbidities. This strict selection limits the generalizability of the findings to a broader DME population. However, despite these positive changes in retinal morphology, no significant improvement in visual acuity was observed. This result is consistent with previous studies suggesting that although reductions in retinal edema are often seen with adjunctive therapies, improvements in visual acuity may not always follow, particularly in patients with chronic or severe DME [33,34,35]. It is possible that the resolution of edema does not immediately translate into functional visual recovery, which may be influenced by factors such as retinal ischemia or permanent damage to the retinal structure [36,37].

Nonetheless, the findings from our study suggest that a fixed combination of bromelain (250 mg) and diosmin mcgSMIN Plus (250 mg) may play an important role in managing DME by helping to control retinal edema between anti-VEGF injections. This could provide a more manageable treatment regimen for patients by ensuring better control of edema during the inter-injection period. Such an approach could lead to decreased healthcare costs, lower patient burden, and fewer clinic visits, which may improve adherence to treatment. Furthermore, adjunctive therapies like this combination may offer a cost-effective means of enhancing patient outcomes while reducing the overall healthcare burden associated with DME management. Potential confounding factors, including variability in glycemic control (HbA1c levels), use of systemic medications (e.g., statins, antihypertensive agents), and lifestyle factors (e.g., smoking, diet, physical activity), were not systematically evaluated during the study and could have influenced treatment outcomes.

Our findings suggest that adjunctive therapy using a fixed combination of bromelain (250 mg) and diosmin mcgSMIN Plus (250 mg) could represent an effective and economically favorable approach to managing DME, potentially reducing the burden on both patients and healthcare systems. Although additional studies are necessary to validate the long-term efficacy and safety of these supplements, the current results offer a foundation for considering their inclusion within a broader DME management framework.

The limitations of the study include sample size, lack of long-term follow-up, and absence of randomization. The relatively small sample size (40 eyes) limits the statistical power and the generalizability of our findings. Larger, multicenter studies are necessary to validate these preliminary results. Moreover, the relatively short follow-up period of four months may not fully capture the long-term effects, safety, or durability of improvements associated with the oral supplement combination. This study focused on clinical and anatomical outcomes; however, we acknowledge that the evaluation of OCT-based biomarkers could provide valuable mechanistic insights into how bromelain and diosmin exert their effects on DME. Future investigations should incorporate these analyses.

## 5. Conclusions

Our results suggest a possible synergistic action of the orally administered combination of Bromelain 250 mg and 250 mg Diosmin mcgSMIN Plus with anti-VEGF intravitreal maintenance therapy in patients affected by cystoid center-involving DME. This combination may provide a more manageable treatment regimen for patients by ensuring better control of edema during the inter-injection period and improving healthcare costs and the workload of healthcare professionals. However, further multicenter, long-term, randomized controlled trials are needed to statistically enhance the understanding of the role of this oral combination in patients treated for DME with anti-VEGF intravitreal maintenance therapy.

## Figures and Tables

**Table 1:** Baseline demographic, clinical, ocular, and systemic characteristics of study patients.

	Total *n* = 40	Group A *n* = 20	Group B *n* = 20	*p*
Age, mean ± SD, years	70 ± 6.77	70.81 ± 7.65	69.11 ± 5.73	0.432
Male, *n* (%)	16 (40)	9 (45)	7 (35)	0.876
BCVA *^1^, mean ± SD, ETDRS letters (Snellen equivalent)	68.26 (20/50) ± 9.22	69.43 (20/40) ± 7.81	68.89 (20/50) ± 8.65	0.579
CRT *^2^, mean ± SD, µm	434.62 ± 47.67	447 ± 58.43	422.25 ± 57.48	0.185

*^1^ BCVA = best-corrected visual acuity; *^2^ CRT = central retinal thickness.

## Data Availability

The original contributions presented in this study are included in the article. Further inquiries can be directed to the corresponding authors.

## Abbreviations

The following abbreviations are used in this manuscript:

CRT	Central retinal thickness
DME	Diabetic macular edema
BCVA	Best-corrected visual acuity
DR	Diabetic retinopathy
VEGF	Vascular endothelial growth factor
SD-OCT	Spectral-domain optical coherence tomography
ELM	External limiting membrane
HF	Hyperreflective foci
